# Programmed death‐ligand 1 expression in swine chronic infections and enhancement of interleukin‐2 production via programmed death‐1/programmed death‐ligand 1 blockade

**DOI:** 10.1002/iid3.510

**Published:** 2021-08-20

**Authors:** Otgontuya Ganbaatar, Satoru Konnai, Tomohiro Okagawa, Yutaro Nojima, Naoya Maekawa, Yoshiki Ichikawa, Atsushi Kobayashi, Tomoyuki Shibahara, Yojiro Yanagawa, Hidetoshi Higuchi, Yukinari Kato, Yasuhiko Suzuki, Shiro Murata, Kazuhiko Ohashi

**Affiliations:** ^1^ Department of Disease Control, Faculty of Veterinary Medicine Hokkaido University Sapporo Japan; ^2^ Department of Advanced Pharmaceutics, Faculty of Veterinary Medicine Hokkaido University Sapporo Japan; ^3^ Department of Veterinary Clinical Medicine, Faculty of Veterinary Medicine Hokkaido University Sapporo Japan; ^4^ Division of Hygiene Management Research National Institute of Animal Health, National Agriculture and Food Research Organization (NARO) Tsukuba Japan; ^5^ Department of Veterinary Science, Graduate School of Life and Environmental Sciences Osaka Prefecture University Osaka Japan; ^6^ Division of Health and Science, School of Veterinary Medicine Rakuno Gakuen University Ebetsu Japan; ^7^ Department of Antibody Drug Development Tohoku University Graduate School of Medicine Sendai Japan; ^8^ Department of Molecular Pharmacology Tohoku University Graduate School of Medicine Sendai Japan; ^9^ Division of Bioresources, International Institute for Zoonosis Control Hokkaido University Sapporo Japan

**Keywords:** interleukin‐2, PD‐1, PD‐L1, swine diseases

## Abstract

**Introduction:**

Chronic infections lead to the functional exhaustion of T cells. Exhausted T cells are phenotypically differentiated by the surface expression of the immunoinhibitory receptor, such as programmed death‐1 (PD‐1). The inhibitory signal is produced by the interaction between PD‐1 and its PD‐ligand 1 (PD‐L1) and impairs the effector functions of T cells. However, the expression dynamics of PD‐L1 and the immunological functions of the PD‐1/PD‐L1 pathway in chronic diseases of pigs are still poorly understood. In this study, we first analyzed the expression of PD‐L1 in various chronic infections in pigs, and then evaluated the immune activation by the blocking assay targeting the swine PD‐1/PD‐L1 pathway.

**Methods:**

In the initial experiments, anti‐bovine PD‐L1 monoclonal antibodies (mAbs) were tested for cross‐reactivity with swine PD‐L1. Subsequently, immunohistochemical analysis was conducted using the anti‐PD‐L1 mAb. Finally, we assessed the immune activation of swine peripheral blood mononuclear cells (PBMCs) by the blockade with anti‐PD‐L1 mAb.

**Results:**

Several anti‐PD‐L1 mAbs tested recognized swine PD‐L1‐expressing cells. The binding of swine PD‐L1 protein to swine PD‐1 was inhibited by some of these cross‐reactive mAbs. In addition, immunohistochemical analysis revealed that PD‐L1 was expressed at the site of infection in chronic infections of pigs. The PD‐L1 blockade increased the production of interleukin‐2 from swine PBMCs.

**Conclusions:**

These findings suggest that the PD‐1/PD‐L1 pathway could be also involved in immunosuppression in chronic infections in pigs. This study provides a new perspective on therapeutic strategies for chronic diseases in pigs by targeting immunosuppressive pathways.

## Graphical abstract

Immunohistochemical staining of PD‐L1 in tissues of pigs with chronic infections. Each section was stained using anti‐bovine PD‐L1 mAb (6C11‐3A11)

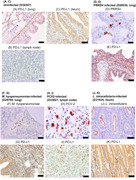

## 1 INTRODUCTION

Programmed death‐ligand‐1 (PD‐L1) is a transmembrane protein that is associated with the suppression of the adaptive immune system. PD‐L1 is expressed in both immune and nonimmune cells, such as antigen‐presenting cells (APCs), lymphocytes, epithelial cells, endothelial cells, and tumor cells.^1^, ^2^, ^3^ Its receptor, PD‐1, is mainly expressed on T lymphocytes in peripheral blood and lymphoid tissues.^1^, ^4^, ^5^ The interaction between PD‐L1 and PD‐1 inhibits T‐cell receptor signaling and suppresses T‐cell effector functions such as cytokine production and cytotoxicity.^1^, ^3^, ^4^, ^5^ During chronic infection, the expression of PD‐1 and PD‐L1 is upregulated and causes the dysfunction of antigen‐specific T cells, which is known as “T‐cell exhaustion.”^4^, ^5^ It has been well documented that T‐cell exhaustion mediated by PD‐1/PD‐L1 contributes to immune evasion in various types of chronic infections by human immunodeficiency virus^5^ and human hepatitis virus type C.^6^ Our previous studies have shown that the PD‐1/PD‐L1 pathway is involved in immune exhaustion and disease progression in chronic infectious diseases of cattle, such as bovine leukemia virus infection,^7^, ^8^, ^9^ Johne's disease,^10^, ^11^ anaplasmosis,^12^ mycoplasmosis,^13^ canine cancers,^14^, ^15^ and equine melanoma.^16^ Furthermore, we established therapeutic antibodies targeting PD‐1 and PD‐L1 and reported their therapeutic efficacy against bovine leukemia virus infection,^17^, ^18^, ^19^ Johne's disease,^20^ mycoplasmosis,^21^ and canine malignant melanoma.^22^, ^23^

Swine PD‐1 and PD‐L1 molecules have been identified, and their amino acid sequences were found to share high identity with human and murine orthologues.^24^, ^25^ The interaction of swine PD‐1 and PD‐L1 decreases the production of interferon‐γ (IFN‐γ) and interleukin‐2 (IL‐2) under T‐cell stimulation in swine peripheral blood mononuclear cells (PBMCs),^24^ indicating the negative regulation of T‐cell response mediated by the PD‐1/PD‐L1 pathway in pigs. During acute infection of classical swine fever virus (CSFV), the expression of *PD‐1* and *PD‐L1* messenger RNA (mRNA) was upregulated in PBMCs, and it was probably correlated with immune inhibition.^26^ In addition, an in vitro model of infection revealed that PD‐L1 expression was increased in dendritic cells infected with porcine circovirus 2 (PCV‐2) and porcine reproductive and respiratory syndrome virus (PRRSV).^27^, ^28^ In this model, gene knockdown of swine PD‐1 decreased apoptosis and increased cell proliferation of swine lymphocytes.^28^ Thus, the swine PD‐1/PD‐L1 pathway is also associated with immune regulation during acute infections of pigs.

However, the involvement of the PD‐1/PD‐L1 pathway in the immunosuppression of chronic infections in pigs has not yet been elucidated, because monoclonal antibody (mAb) against swine PD‐1 and PD‐L1, which could be powerful tools for elucidating the pathogenesis of swine diseases, have not yet been reported. In this study, we verified the cross‐reactivity of our established anti‐bovine PD‐L1 mAbs with swine PD‐L1. Then, we performed the immunohistochemical analysis in various swine chronic infections using the anti‐PD‐L1 mAb. Finally, we evaluated the immune activation of swine PBMCs by blockade with the anti‐PD‐L1 mAb.

## 2 MATERIALS AND METHODS

### 2.1 Isolation of swine PBMCs

Heparin‐treated blood samples were collected from piglets (crossbreed, large white × Landrace × large white × Duroc, 1‐ to 6‐months‐old, male or female) raised on conventional farms in Hokkaido, Japan. PBMCs were separated from blood samples by density gradient centrifugation using Percoll (GE Healthcare). All experimental procedures were carried out with the approval of the Animal Experiment Committee of Hokkaido University (20‐0093). Informed consent was obtained from all owners.

### 2.2 Preparation of swine PD‐1‐ and PD‐L1‐expressing cells

The complementary DNA (cDNA) was synthesized from mRNA of swine PBMCs stimulated with 20 ng/ml phorbol 12‐myristate acetate (PMA; Sigma‐Aldrich) and 1 μg/ml ionomycin (Sigma‐Aldrich) for 24 h. The cultivation of PBMCs, total RNA isolation, and cDNA synthesis was conducted as described previously.^16^ The cDNAs encoding swine PD‐1 and PD‐L1 were amplified by PCR using TaKaRa Ex Taq (Takara Bio) and gene‐specific primers with restriction enzyme cleavage sites (Table S1), and then subcloned into pEGFP‐N2 (Clontech). The purified plasmids were transfected to COS‐7 cells using Lipofectamine 3000 Reagent (Thermo Fisher Scientific). After 48 h of cell cultivation, the localization of PD‐1‐EGFP and PD‐L1‐EGFP in the cells was confirmed using ZOE Fluorescent Cell Imager (Bio‐Rad).

### 2.3 Preparation of recombinant swine PD‐1 and PD‐L1

The cDNAs encoding the signal sequences and extracellular domain fragments of swine PD‐1 and PD‐L1 were amplified by PCR using gene‐specific primers with restriction enzyme cleavage sites (Table S1). The amplified products were subcloned with a gene cassette encoding the Fc region of rabbit IgG at the multi‐cloning site of pCXN2.1(+) (kindly provided by Dr. T. Yokomizo, Juntendo University, Japan). Recombinant swine PD‐1 and PD‐L1 proteins (rPD‐1 and rPD‐L1) were produced using the Expi293 Expression System (Thermo Fisher Scientific) and purified from the culture supernatants by affinity chromatography using Ab‐Capcher ExTra (ProteNova). The purity of the proteins was evaluated by sodium dodecyl sulfate‐polyacrylamide gel electrophoresis (SDS‐PAGE) in reducing or nonreducing conditions using SuperSep Ace 5%–20% gradient polyacrylamide gel (FUJIFILM Wako Pure Chemical). The protein concentration was measured by ultraviolet absorbance at 280 nm with a NanoDrop 8000 Spectrophotometer (Thermo Fisher Scientific). The binding of rPD‐1 and rPD‐L1 to COS‐7 cells expressing swine PD‐L1‐EGFP and PD‐1‐EGFP was investigated using flow cytometry as described previously.^16^

### 2.4 Cross‐reactivity assay against swine PD‐L1

The cross‐reactivity of anti‐bovine PD‐L1 mAbs to swine PD‐L1 was examined by flow cytometry as described previously with modifications.^16^ Briefly, swine PD‐L1‐EGFP‐expressing cells were incubated with rat anti‐bovine PD‐L1 mAbs (4G12‐C1, 5A2‐A1, 6C11‐3A11, and 6G7‐E1) for 20 min at 25°C. Rat immunoglobulin G 1 (IgG_1_), IgG_2a_, and IgM isotype controls (BD Biosciences) were used as isotype‐matched negative controls. The details of the primary antibodies used in this assay are shown in Table S2. The cells were washed with PBS containing 1% bovine serum albumin (Sigma‐Aldrich) and then stained with APC‐conjugated goat anti‐rat immunoglobulin antibody (Southern Biotech) for 20 min at 25°C. After washing, the stained cells were examined by FACS Verse (BD Biosciences). In addition, the binding ability to swine PBMCs was also evaluated. Briefly, PBMCs were cultivated with 10 μg/ml swine IFN‐γ (Kingfisher Biotech) or 20 ng/ml PMA (Sigma‐Aldrich) and 1 μg/ml ionomycin (Sigma‐Aldrich). To prevent nonspecific reactions, the stimulated PBMCs were incubated with PBS supplemented with 10% goat serum (Thermo Fisher Scientific) for 15 min at 25°C, and cells were stained as described above.

### 2.5 Blockade assay of swine PD‐1/PD‐L1 binding

To confirm the ability of anti‐PD‐L1 mAbs to interrupt swine PD‐1/PD‐L1 binding, blocking assays were conducted using swine rPD‐1 and rPD‐L1 as described previously with modifications.^16^ Briefly, anti‐PD‐L1 mAbs (4G12‐C1, 5A2‐A1, 6C11‐3A11, and 6G7‐E1) or rat IgG_1_, IgG_2a_, and IgM isotype controls (BD Biosciences) (Table S2) were preincubated with biotinylated rPD‐L1 at various concentrations for 30 min at 37°C. The preincubated reagents were added to 96‐well microplates coated with rPD‐1 (1 μg/ml) and incubated at 37°C for 30 min. The binding of rPD‐L1 was determined using Neutravidin conjugated with horseradish peroxidase (Thermo Fisher Scientific) and TMB One Component Substrate (Bethyl Laboratories). The optical density at 450 nm (OD450) was measured with an MTP‐900 microplate reader (Corona Electric). Three independent experiments were performed in duplicate. The relative binding of rPD‐L1 to rPD‐1 was calculated from OD450 of sample preincubated with each antibody, using that preincubated without antibody as 100%.

### 2.6 Immunohistochemical assays

Tissue specimens of pigs were collected for pathological diagnosis at the National Institute of Animal Health, National Agriculture, and Food Research Organization, Japan. These pigs were confirmed to be infected with PRRSV, *Mycoplasma hyopneumoniae*, PCV‐2, and *Lawsonia intracellularis*, respectively, by the immunohistochemistry as shown below and showed typical clinical signs and pathological lesions. Tissue samples from a healthy pig were confirmed as being not infected with these pathogens and were used as a negative control. Detailed information on tissue specimens is shown in Table S3.

Immunohistochemistry was performed to detect the antigens of PRRSV, *M. hyopneumoniae*, PCV‐2, and *L. intracellularis*. Formalin‐fixed, paraffin‐embedded tissues were cut into 3‐μm‐thick sections and incubated with 0.3% hydrogen peroxide in methanol to inhibit the activity of endogenous peroxidase. Antigen retrieval was performed as shown in Table S4. The section was incubated with each primary antibody as shown in Table S4 and was followed by a secondary antibody (Histofine Simple Stain MAX‐PO Mouse or Multi) (Nichirei Bioscience). The sections were then incubated with Histofine Simple Stain AEC solution (Nichirei Bioscience). All immunostained sections were observed under an optical microscope.

For the immunohistochemical assay of PD‐L1, the tissues were fixed in formalin, embedded in paraffin wax, and cut into 4‐μm‐thick sections. The sections were autoclaved in 10 mM EDTA for antigen retrieval and then incubated with 0.3% hydrogen peroxide in methanol to inhibit the activity of endogenous peroxidase. The sections were incubated overnight at 4°C with or without anti‐PD‐L1 mAb (6C11‐3A11) (Table S2) and then detected using the Vectastain Elite ABC Rat IgG kit (Vector Laboratories). The reaction was visualized using 3,3ʹ‐diaminobenzidine tetrahydrochloride. All immunostained sections were observed under an optical microscope.

### 2.7 Immunoactivation assay

PBMCs were isolated from the peripheral blood of six healthy piglets (crossbreed, large white × Landrace × large white × Duroc, 7‐week‐old, male or female). To examine the effects of the inhibition of the PD‐1/PD‐L1 pathway on swine immune cells, PBMCs (2 × 10^6^ cells/ml) were cultured with 0.1 μg/ml of *Staphylococcal* Enterotoxin B (Sigma‐Aldrich) in the presence of 10 μg/ml of anti‐PD‐L1 mAb (4G12‐C1) or rat IgG_2a_ control (2A3; Bio X Cell) (Table S2) in RPMI 1640 medium (Sigma‐Aldrich) supplemented with 10% heat‐inactivated fetal bovine serum (Thermo Fisher Scientific), 2 mM ‐L‐glutamine, 100 U/ml penicillin, and 100 μg/ml streptomycin (Thermo Fisher Scientific) at 37°C, 5% CO_2_ for 3 days. After the cultivation, IL‐2 levels in the culture supernatant were assessed in duplicate by the Swine IL‐2 DuoSet enzyme‐linked immunosorbent assay (ELISA) development kit (R&D Systems) according to the manufacturer's recommendations.

### 2.8 Statistical analysis

Differences were identified using the Wilcoxon signed‐rank test. All statistical tests were performed using the statistical analysis program MEPHAS (http://www.gen-info.osaka-u.ac.jp/MEPHAS/). *p* <0.05 were considered significant.

## 3 RESULTS

### 3.1 Confirmation of cellular localization and binding of swine PD‐1 and PD‐L1

**Figure 1:**
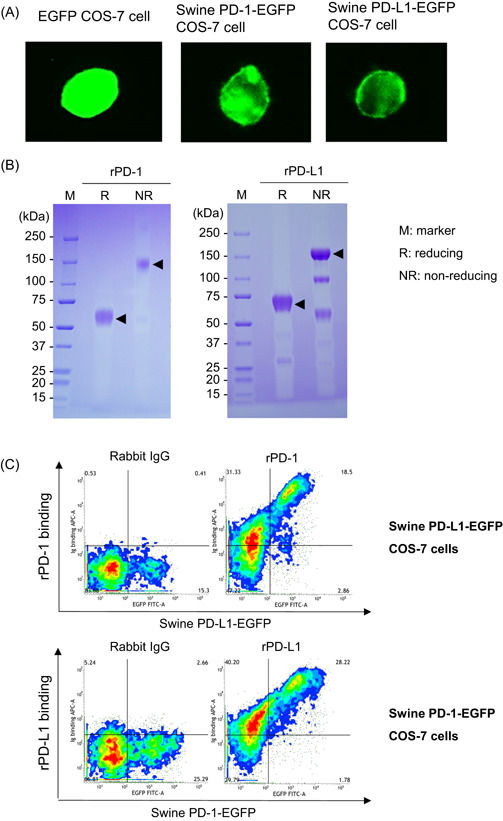
Confirmation of binding ability of swine PD‐1 and PD‐L1. (A) COS‐7 cells expressing EGFP (control, left panel), swine PD‐1‐EGFP (middle panel), or PD‐L1‐EGFP (right panel). The localization of EGFP, PD‐1‐EGFP, and PD‐L1‐EGFP in the cells was confirmed by fluorescence microscopy. (B) rPD‐1 (left panel) and rPD‐L1 (right panel) were produced using the Expi293 Expression System. These proteins were purified from culture supernatants and analyzed using SDS‐PAGE. (C) Binding ability of rPD‐1 (upper panel) or rPD‐L1 (lower panel) to cells expressing swine PD‐L1‐EGFP or PD‐1‐EGFP. The binding of Ig fusion proteins was analyzed by flow cytometry. EGFP, enhanced green fluorescent protein; IgG, immunoglobulin G; PD‐1, programmed death‐1; PD‐L1, programmed death‐ligand 1; rPD‐1, recombinant PD‐1; SDS‐PAGE, sodium dodecyl sulfate‐polyacrylamide gel electrophoresis

We first analyzed the cellular localization of swine PD‐1‐EGFP and PD‐L1‐EGFP proteins in the overexpressed COS‐7 cell lines by fluorescence microscopy. Swine PD‐1‐EGFP and PD‐L1‐EGFP were localized on the cell surface of the overexpressed cell lines (Figure 1A). Soluble rPD‐1 and rPD‐L1 were produced in Expi293F cells and purified from the culture supernatant with protein A resin (Figure 1B). They were dimerized by disulfide bonds (Figure 1B). rPD‐1 and rPD‐L1 bound to the cells expressing swine PD‐L1‐EGFP and PD‐1‐EGFP, respectively (Figure 1C).

### 3.2 Binding ability of anti‐bovine PD‐L1 mAbs to swine PD‐L1

**Figure 2:**
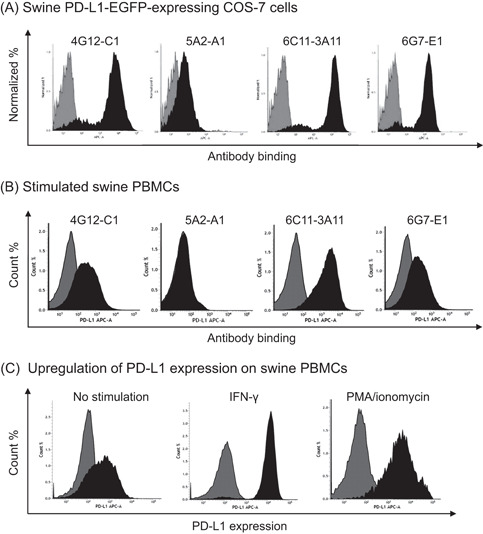
Binding ability of anti‐bovine PD‐L1 mAbs against swine PD‐L1. Binding activities of anti‐PD‐L1 mAbs to (A) swine PD‐L1‐EGFP overexpressed in COS‐7 cells and (B) swine PBMC stimulated with PMA/ionomycin. (C) Expression analysis of swine PD‐L1 on PBMCs was performed using an anti‐bovine PD‐L1 mAb (6C11‐3A11) by flow cytometry. APC‐conjugated anti‐rat Ig antibody was used to label the primary mAbs and analyzed. Rat IgG_1_, IgG_2a_, and IgM controls were used as isotype‐matched negative controls. Binding activities of anti‐PD‐L1 mAbs and the negative controls were shown by black and gray histograms, respectively. EGFP, enhanced green fluorescent protein; IFN‐γ, interferon‐γ; Ig, immunoglobulin; mAb, monoclonal antibody; PD‐1, programmed death‐1; PD‐L1, programmed death‐ligand 1; PMA, phorbol 12‐myristate acetate; PMBC, peripheral blood mononuclear cell

Anti‐bovine PD‐L1 mAbs were tested for cross‐reactivity with swine PD‐L1. All tested mAbs detected swine PD‐L1‐EGFP in the overexpressed cell line (Figure 2A). In addition, anti‐PD‐L1 mAbs, except for 5A2‐A1, detected PD‐L1 in swine PBMCs stimulated with PMA/ionomycin (Figure 2B). The mAb 6C11‐3A11 showed was the strongest binding intensity to the overexpressing COS‐7 cells and stimulated PBMCs. Thus, PD‐L1 expression was further analyzed in swine PBMCs under IFN‐γ stimulation using the mAb 6C11‐3A11. Stimulation with IFN‐γ strikingly upregulated PD‐L1 expression on swine PBMCs (Figure 2C).

### 3.3 Inhibition of swine PD‐1/PD‐L1 binding by anti‐PD‐L1 mAbs

**Figure 3:**
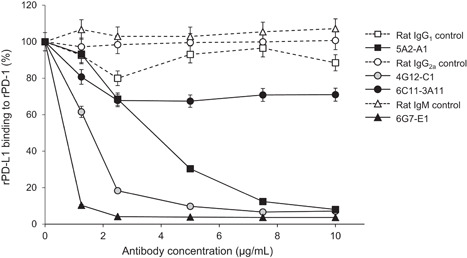
Blockade of swine PD‐1/PD‐L1 binding by anti‐PD‐L1 mAbs. Preincubated anti‐PD‐L1 mAbs or rat isotype controls with biotinylated rPD‐L1 were added to 96‐well microplates coated with rPD‐1 and incubated at 37°C for 30 min. Each curve represents the relative binding of rPD‐L1 preincubated with antibodies compared with that preincubated with no antibody. Each point represents the mean of three independent experiments. IgG, immunoglobulin G; mAb, monoclonal antibody; PD‐1, programmed death‐1; PD‐L1, programmed death‐ligand 1; rPD‐1, recombinant PD‐1

To determine whether anti‐bovine PD‐L1 mAbs are capable of inhibiting the swine PD‐1/PD‐L1 binding, we performed an ELISA with rPD‐1 and rPD‐L1 in the presence of anti‐PD‐L1 mAbs. All of the tested anti‐PD‐L1 mAbs blocked the binding of rPD‐L1 to rPD‐1 (Figure 3). The blocking activity was strong in the order of 6G7‐E1, 4G12‐C1, 5A2‐A1, and 6C11‐3A11.

### 3.4 PD‐L1 expression in the tissue of several disease‐infected pigs

**Figure 4:**
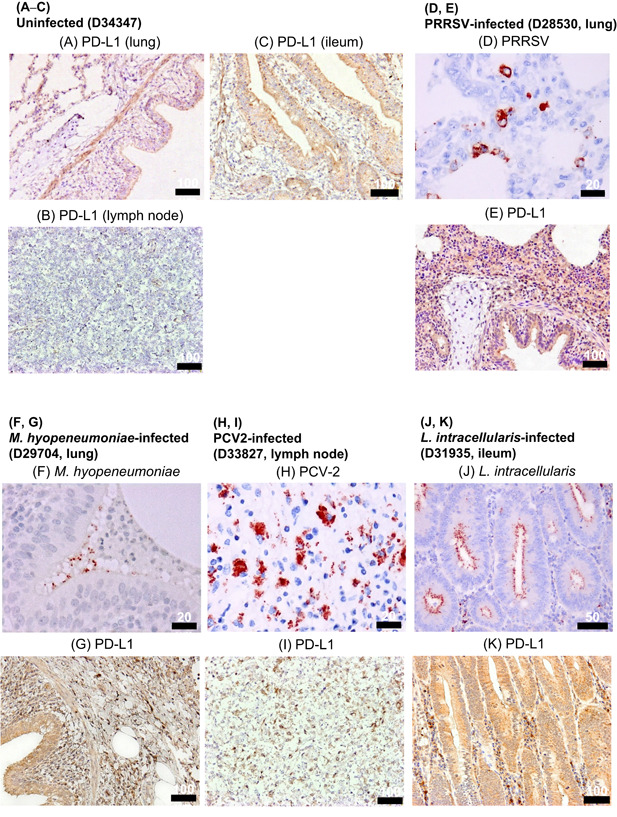
Immunohistochemical analysis of PD‐L1 in swine chronic infections. (A–C, E, G, I, K) Immunohistochemical staining of PD‐L1 in tissues of pigs with chronic infections (PRRS, mycoplasmosis, PCV2‐infection, *Lawsonia intracellularis* infection). Each section was stained using anti‐bovine PD‐L1 mAb (6C11‐3A11). Matched tissue sections stained without a primary antibody as negative controls are shown in Figure S1. (D, F, H, J) Antigens of PRRSV, *Mycoplasma hyopneumoniae*, PCV2, and *L. intracellularis* were stained with specific antibodies in the tissue sections. mAb, monoclonal antibody; PCV2, porcine circovirus 2; PD‐L1, programmed death‐ligand 1; PRRS, porcine reproductive and respiratory syndrome; PRRS, PRRS, virus

In pigs infected with PRRSV, *M. hyopneumoniae*, PCV‐2, and *L. intracellularis*, PD‐L1 expression was evaluated at the site of infection and compared with uninfected tissues from a healthy pig. The antigens of PRRSV, *M. hyopneumoniae*, PCV‐2, and *L. intracellularis* were detected in the tissues of the infected animals, respectively, but not in the tissues of uninfected control (Figure 4D,F,H,J). In animal D28530, PRRSV was detected in the cytoplasm of macrophages in the lung interstitium (Figure 4D). In the lung of animal D29704, *M. hyopneumoniae* was detected on the microvilli of bronchial epithelial cells (Figure 4F). In animal D33827, PCV‐2 was detected in intracytoplasmic inclusion bodies of macrophages in the lymph node (Figure 4H). In animal D31935, *L. intracellularis* was detected in the cytoplasm of intestinal epithelial cells (Figure 4J). PD‐L1 was strongly expressed in the lung of PRRSV‐ or *M. hyopneumoniae*‐infected pig, the mesenteric lymph nodes of PCV‐2‐infected pig, and the ileum of a pig infected with *L. intracellularis* (Figure 4E,G,I,K). In the uninfected controls, the mucosal epithelium and submucosal muscular plate of ileum and tracheal and bronchial epithelium and alveolar walls of lungs were stained very lightly for PD‐L1 (Figure 4A,C).

### 3.5 Immune activation in swine PBMCs by PD‐L1 blockade

**Figure 5:**
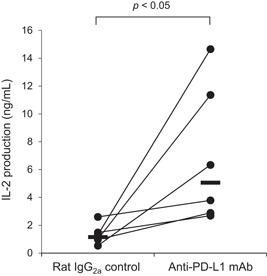
IL‐2 production via PD‐L1 blockade by anti‐PD‐L1 mAb. PBMCs were isolated from healthy piglets (*n* = 6) and cultured with anti‐PD‐L1 mAb (4G12‐C1) or rat IgG_2a_ control in the presence of SEB. After 3 days, culture supernatants were collected and IL‐2 levels were measured by ELISA in duplicate. Significant differences between the treatments were identified using the Wilcoxon signed‐rank test. ELISA, enzyme‐linked immunosorbent assay; IgG, immunoglobulin G; IL, interleukin; mAb, monoclonal antibody; PD‐L1, programmed death‐ligand 1; PMBC, peripheral blood mononuclear cell; SEB, *Staphylococcal* enterotoxin B

Finally, the effect of PD‐L1 blockade on immune activation was evaluated by the change in IL‐2 production in swine PBMCs. Blocking with anti‐PD‐L1 mAb (4G12‐C1) significantly increased IL‐2 production from all swine PBMCs, although individual differences were observed (Figure 5).

## 4 DISCUSSION

The immune system plays a critical role in the response to chronic infection, but viruses and bacteria have evolved some strategies to interfere with host immunity. Some of the pathogens evade host immune responses and develop a persistent infection, resulting in the persistent stimulation of antigens and progressive T‐cell dysfunction.^4^, ^5^ During chronic infections, the PD‐1/PD‐L1 pathway has been shown to mediate T‐cell dysfunction and limit pathogen clearance.

Because of the lack of available treatment, there are still many chronic infectious diseases with poor prognosis in domestic animals, including pigs. Moreover, the lack of a better understanding of the immunological pathways leading to immune evasion can be attributed to this process. To date, only a few studies have shown that the expression of PD‐1 and PD‐L1 is significantly increased in immune cells of pigs during acute infection caused by CSFV or PCV‐2^26^, ^29^ or in the cell culture experiments with PCV‐2 and/or PRRSV.^27^, ^28^ In this study, we focused on chronic infections of pigs and found that PD‐L1 expression was also upregulated in infected lesions of pigs affected with PRRS, mycoplasmosis, PCV‐2 infection, and proliferative enteropathy. Previous studies have reported the suppression of the T‐cell response in pigs with these chronic infections.^30^, ^31^, ^32^, ^33^ Further studies are needed to elucidate detailed mechanisms of the suppression of T‐cell response mediated by PD‐1/PD‐L1 in these diseases. Furthermore, IFN‐γ stimulation strongly induced the upregulation of PD‐L1 expression in PBMCs. These results suggest that inflammatory cytokines can regulate the expression of PD‐L1 in swine immune cells. Unfortunately, our previously established anti‐bovine PD‐1 mAbs^8^ did not cross‐react with swine PD‐1, thus we were not able to determine the trend of PD‐1 expression in these swine infections (data not shown). Analysis of PD‐1 expression during disease progression will be also helpful for understanding the immunopathogenesis of chronic infections in pigs. The relationship between PD‐1 and suppression of T‐cell responses in these diseases also needs to be clarified and further studies are needed.

In this study, we compared the cross‐reactivity to swine PD‐L1 and the blockade activity against the interaction of swine PD‐1/PD‐L1 among four clones of anti‐bovine PD‐L1 mAbs. The mAb 6C11‐3A11 exerted high binding affinity to swine PD‐L1 and was found to be the optimal detection antibody for PD‐L1 in pigs. However, it had limited blockade activity. On the contrary, the mAbs 6G7‐E1 and 4G12‐C1 showed moderate binding affinity to swine PD‐L1, but their blockade activities were robust. The difference among these three mAbs would depend on the epitope of the mAbs. The mAbs 4G12‐C1 and 6G7‐E1 may interact with the region of swine PD‐L1 which is essential for the interaction with swine PD‐1. The subclass of the mAb 4G12‐C1 is IgG2a, and this clone is considered to be suitable for the future development of chimeric antibodies with the swine IgG subclass. PD‐L1 blockade using the mAb 4G12‐C1 increased the production of IL‐2 from swine PBMCs. IL‐2 is produced mainly by activated T cells and is vital for the cellular expansion required for functional immune response. Moreover, the immune activation effect by the PD‐L1 blockade, such as the production of other Th1 cytokines and proliferative and effector activities of antigen‐specific T cells, must be further analyzed in pigs. The antibacterial and antiviral effects of the PD‐L1 blockade will be tested in clinical studies using infected animals. Thus, to develop a new therapeutic method for the control of chronic diseases in pigs, the effect of the PD‐L1 blockade must be evaluated from a variety of perspectives.

## CONFLICT OF INTERESTS

The authors declare that there are no conflict of interests.

## AUTHOR CONTRIBUTIONS

Satoru Konnai, Tomohiro Okagawa, Naoya Maekawa, Shiro Murata, and Kazuhiko Ohashi designed the work. Otgontuya Ganbaatar, Tomohiro Okagawa, Yutaro Nojima, and Yoshiki Ichikawa performed the experiments. Atsushi Kobayashi, Tomoyuki Shibahara, Yojiro Yanagawa, Hidetoshi Higuchi, Yukinari Kato, and Yasuhiko Suzuki provided intellectual input, field samples, laboratory materials, reagents, and/or analytic tools. Otgontuya Ganbaatar, Satoru Konnai, Tomohiro Okagawa, and Atsushi Kobayashi acquired, analyzed, and interpreted the data. Otgontuya Ganbaatar, Satoru Konnai, and Tomohiro Okagawa wrote the manuscript. Satoru Konnai, Tomohiro Okagawa, Naoya Maekawa, Atsushi Kobayashi, Tomoyuki Shibahara, Yojiro Yanagawa, Hidetoshi Higuchi, Yukinari Kato, Yasuhiko Suzuki, Shiro Murata, Kazuhiko Ohashi revised the manuscript. All authors read and approved the final manuscript.

## Supporting information

Supporting information.

## Data Availability

The data that support the findings of this study are available from the corresponding author upon reasonable request.
