# Supplementary material for: Programmed death‐ligand 1 expression in swine chronic infections and enhancement of interleukin‐2 production via programmed death‐1/programmed death‐ligand 1 blockade
Source: Immun Inflamm Dis. 2021 Aug 20;9(4):1573–83. doi: 10.1002/iid3.510 (PMC8589367; doi:10.1002/iid3.510)
Supplement: Supplementary file 1 — Supporting information. [file IID3-9-1573-s001.pptx]

## Slide 1
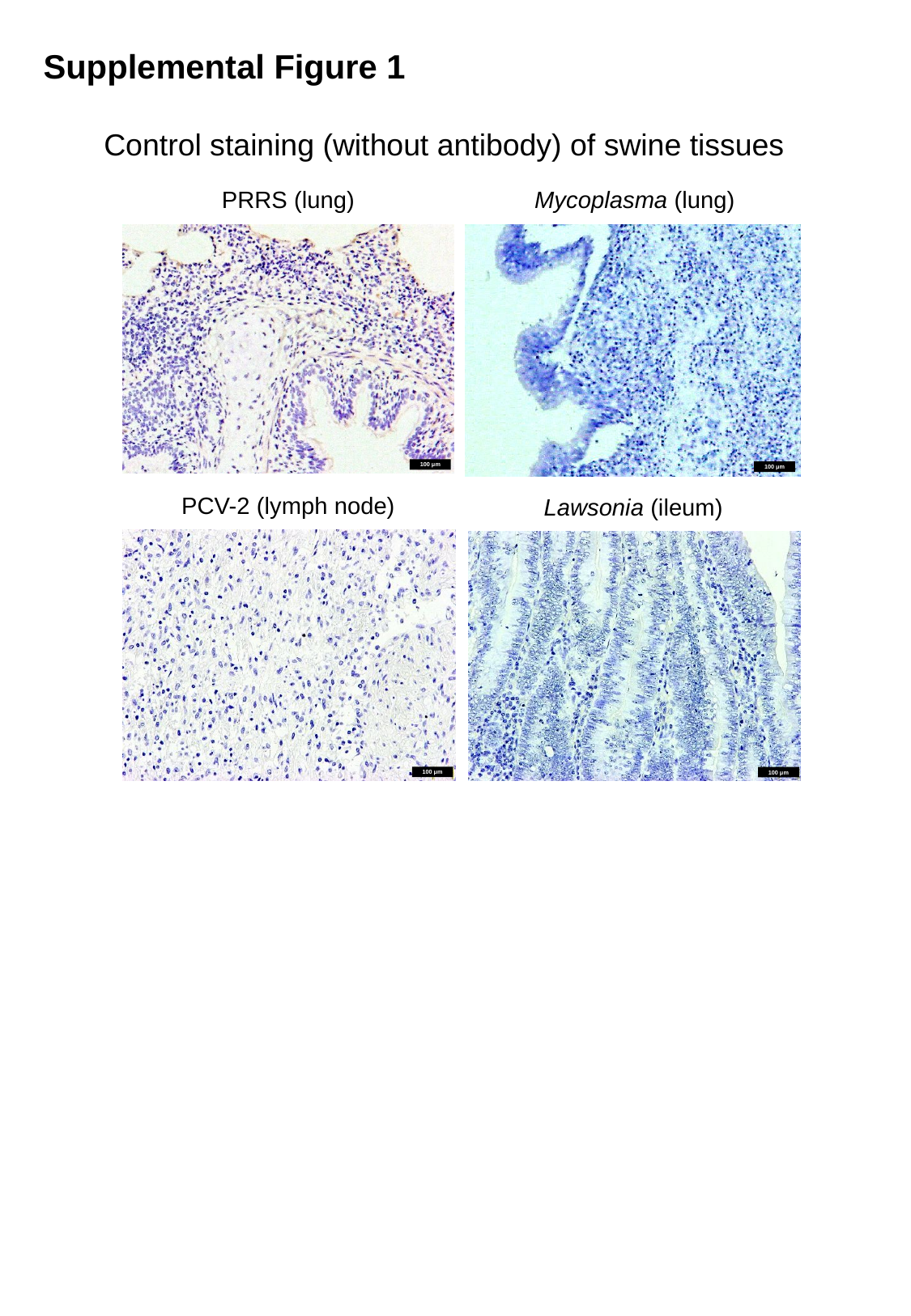

# Supplemental Figure 1
Control staining (without antibody) of swine tissues
PRRS (lung)
Mycoplasma (lung)
PCV-2 (lymph node)
Lawsonia (ileum)
100 μm
100 μm
100 μm
100 μm

## Slide 2
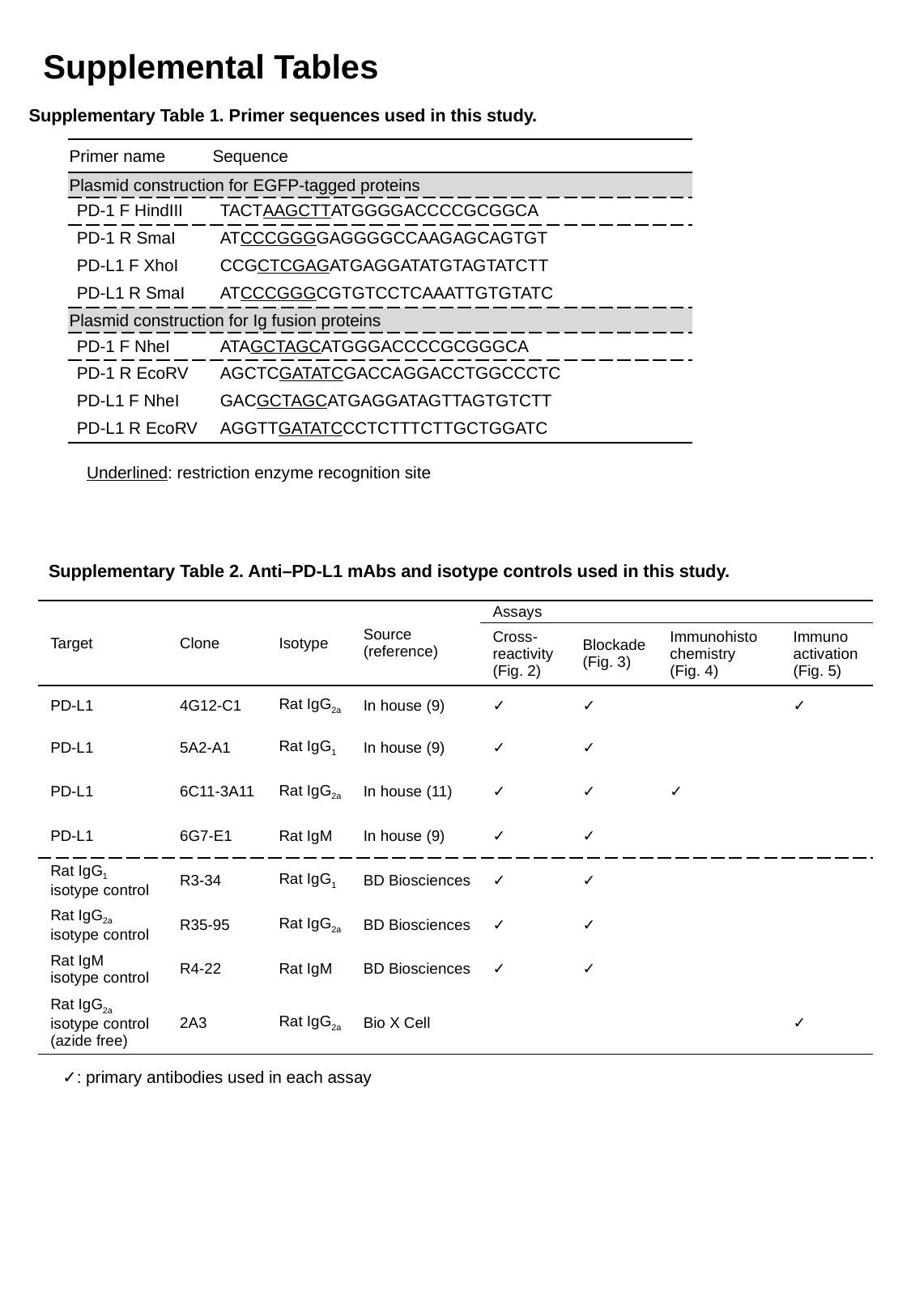

Supplemental Tables
Supplementary Table 1. Primer sequences used in this study.
| Primer name | Sequence |
| --- | --- |
| Plasmid construction for EGFP-tagged proteins | |
| PD-1 F HindIII | TACTAAGCTTATGGGGACCCCGCGGCA |
| PD-1 R SmaI | ATCCCGGGGAGGGGCCAAGAGCAGTGT |
| PD-L1 F XhoI | CCGCTCGAGATGAGGATATGTAGTATCTT |
| PD-L1 R SmaI | ATCCCGGGCGTGTCCTCAAATTGTGTATC |
| Plasmid construction for Ig fusion proteins | |
| PD-1 F NheI | ATAGCTAGCATGGGACCCCGCGGGCA |
| PD-1 R EcoRV | AGCTCGATATCGACCAGGACCTGGCCCTC |
| PD-L1 F NheI | GACGCTAGCATGAGGATAGTTAGTGTCTT |
| PD-L1 R EcoRV | AGGTTGATATCCCTCTTTCTTGCTGGATC |
Underlined: restriction enzyme recognition site
Supplementary Table 2. Anti–PD-L1 mAbs and isotype controls used in this study.
| Target | Clone | Isotype | Source (reference) | Assays | | | |
| --- | --- | --- | --- | --- | --- | --- | --- |
| | | | | Cross- reactivity (Fig. 2) | Blockade (Fig. 3) | Immunohisto chemistry (Fig. 4) | Immuno activation (Fig. 5) |
| PD-L1 | 4G12-C1 | Rat IgG2a | In house (9) | ✓ | ✓ | | ✓ |
| PD-L1 | 5A2-A1 | Rat IgG1 | In house (9) | ✓ | ✓ | | |
| PD-L1 | 6C11-3A11 | Rat IgG2a | In house (11) | ✓ | ✓ | ✓ | |
| PD-L1 | 6G7-E1 | Rat IgM | In house (9) | ✓ | ✓ | | |
| Rat IgG1 isotype control | R3-34 | Rat IgG1 | BD Biosciences | ✓ | ✓ | | |
| Rat IgG2a isotype control | R35-95 | Rat IgG2a | BD Biosciences | ✓ | ✓ | | |
| Rat IgM isotype control | R4-22 | Rat IgM | BD Biosciences | ✓ | ✓ | | |
| Rat IgG2a isotype control (azide free) | 2A3 | Rat IgG2a | Bio X Cell | | | | ✓ |
✓: primary antibodies used in each assay

## Slide 3
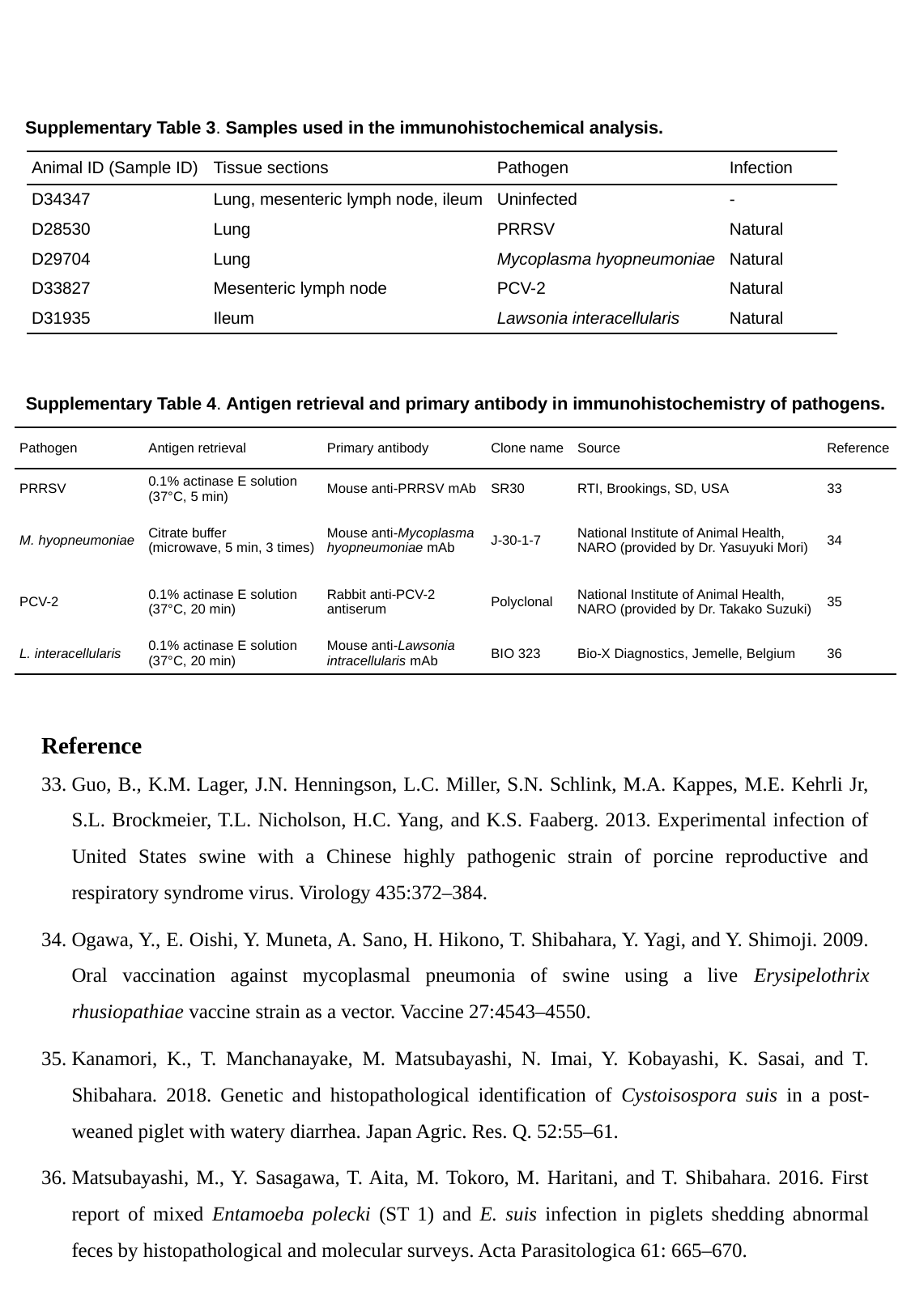

Supplementary Table 3. Samples used in the immunohistochemical analysis.
| Animal ID (Sample ID) | Tissue sections | Pathogen | Infection |
| --- | --- | --- | --- |
| D34347 | Lung, mesenteric lymph node, ileum | Uninfected | - |
| D28530 | Lung | PRRSV | Natural |
| D29704 | Lung | Mycoplasma hyopneumoniae | Natural |
| D33827 | Mesenteric lymph node | PCV-2 | Natural |
| D31935 | Ileum | Lawsonia interacellularis | Natural |
Supplementary Table 4. Antigen retrieval and primary antibody in immunohistochemistry of pathogens.
| Pathogen | Antigen retrieval | Primary antibody | Clone name | Source | Reference |
| --- | --- | --- | --- | --- | --- |
| PRRSV | 0.1% actinase E solution (37°C, 5 min) | Mouse anti-PRRSV mAb | SR30 | RTI, Brookings, SD, USA | 33 |
| M. hyopneumoniae | Citrate buffer (microwave, 5 min, 3 times) | Mouse anti-Mycoplasma hyopneumoniae mAb | J-30-1-7 | National Institute of Animal Health, NARO (provided by Dr. Yasuyuki Mori) | 34 |
| PCV-2 | 0.1% actinase E solution (37°C, 20 min) | Rabbit anti-PCV-2 antiserum | Polyclonal | National Institute of Animal Health, NARO (provided by Dr. Takako Suzuki) | 35 |
| L. interacellularis | 0.1% actinase E solution (37°C, 20 min) | Mouse anti-Lawsonia intracellularis mAb | BIO 323 | Bio-X Diagnostics, Jemelle, Belgium | 36 |
Reference
Guo, B., K.M. Lager, J.N. Henningson, L.C. Miller, S.N. Schlink, M.A. Kappes, M.E. Kehrli Jr, S.L. Brockmeier, T.L. Nicholson, H.C. Yang, and K.S. Faaberg. 2013. Experimental infection of United States swine with a Chinese highly pathogenic strain of porcine reproductive and respiratory syndrome virus. Virology 435:372–384.
Ogawa, Y., E. Oishi, Y. Muneta, A. Sano, H. Hikono, T. Shibahara, Y. Yagi, and Y. Shimoji. 2009. Oral vaccination against mycoplasmal pneumonia of swine using a live Erysipelothrix rhusiopathiae vaccine strain as a vector. Vaccine 27:4543–4550.
Kanamori, K., T. Manchanayake, M. Matsubayashi, N. Imai, Y. Kobayashi, K. Sasai, and T. Shibahara. 2018. Genetic and histopathological identification of Cystoisospora suis in a post-weaned piglet with watery diarrhea. Japan Agric. Res. Q. 52:55–61.
Matsubayashi, M., Y. Sasagawa, T. Aita, M. Tokoro, M. Haritani, and T. Shibahara. 2016. First report of mixed Entamoeba polecki (ST 1) and E. suis infection in piglets shedding abnormal feces by histopathological and molecular surveys. Acta Parasitologica 61: 665–670.
